# Genetic profile of Egyptian hepatocellular-carcinoma associated with hepatitis C virus Genotype 4 by 15 K cDNA microarray: Preliminary study

**DOI:** 10.1186/1756-0500-1-106

**Published:** 2008-10-29

**Authors:** Abdel-Rahman N Zekri, Mohamed M Hafez, Abeer A Bahnassy, Zeinab K Hassan, Tarek Mansour, Mahmoud M Kamal, Hussein M Khaled

**Affiliations:** 1 Virology and Immunology Unit, Cancer Biology Department, National Cancer Institute, Cairo University, 1st Kasr El-Aini st, Cairo, Egypt; 2 Tissue Culture Unite Pathology Department, National Cancer Institute, Cairo University, Cairo, Egypt; 3 Clinical Pathology Department, National Cancer Institute, Cairo University, Cairo, Egypt; 4 Medical Oncology Department, National Cancer Institute, Cairo University, Cairo, Egypt

## Abstract

**Background:**

Hepatocellular carcinoma (HCC) is a preventable disease rather than a curable one, since there is no well-documented effective treatment modality until now, making the molecular study of this disease mandatory.

**Findings:**

We studied gene expression profile of 17 Egyptian HCC patients associated with HCV genotype-4 infection by c-DNA microarray. Out of the 15,660 studied genes, 446 were differentially expressed; 180 of them were up regulated and 134 were down regulated. Seventeen genes out of the 180 up-regulated genes are involved in 28 different pathways. Protein phosphatase 3 (PPP3R1) is involved in 10 different pathways followed by fibroblast growth factor receptor 1 (FGFR1), Cas-Br-M ecotropic retroviral transforming sequence b (CBLB), spleen tyrosine kinase (SYK) involved in three pathways; bone morphogenetic protein 8a (BMP8A), laminin alpha 3 (LAMA3), cell division cycle 23 (CDC23) involved in 2 pathways and NOTCH4 which regulate Notch signaling pathway. On the other hand, 25 out of the 134 down-regulated genes are involved in 20 different pathways. Integrin alpha V alpha polypeptide antigen CD51 (ITGVA) is involved in 4 pathways followed by lymphotoxin alpha (TNF superfamily, member 1) (LTA) involved in 3 pathways and alpha-2-macroglobulin (A2M), phosphorylase kinase alpha 2-liver (PHKA2) and MAGI1 membrane associated guanylate kinase 1 (MAGI1) involved in 2 pathways. In addition, 22 genes showed significantly differential expression between HCC cases with cirrhosis and without cirrhosis. Confirmation analysis was performed on subsets of these genes by RT-PCR, including some up-regulated genes such as CDK4, Bax, NOTCH4 and some down-regulated genes such as ISGF3G, TNF, and VISA.

**Conclusion:**

This is the first preliminary study on gene expression profile in Egyptian HCC patients associated with HCV-Genotype-4 using the cDNA microarray. The identified genes could provide a new gate for prognostic and diagnostic markers for HCC associated with HCV. They could also be used to identify candidate genes for molecular target therapy.

## Background

Hepatocellular carcinoma (HCC) is one of the most malignant tumors with a high mortality, aggressive growth behavior and a high recurrence rate. It is the sixth most common cancer worldwide and the third most common cause of cancer death with prevalent areas in Asia and sub-Saharan Africa [1]. HCC usually develops following chronic liver inflammation caused by hepatitis C or B virus [2]. Although recent studies showed increased HCC incidence in western countries, more than 80% of cases occurred in endemic areas due to exposure to hepatitis viruses, mycotoxins and alcohol abuse [3]. Since HCC progression is usually asymptomatic and results in poor prognosis with low 5-year survival rates (12–15%), comprehensive molecular genetic studies will be important for improving clinical management of HCC.

The major etiological factor of liver cancer is hepatitis B virus (HBV), followed by hepatitis C virus infection (HCV). Although HCC tissue from different individuals has many phenotypic differences, there are some features that unify HCC occurring in a background of viral hepatitis B and C. HCC due to HBV and/or HCV may be an indirect effect of enhanced hepatocyte turnover, which occurs in order to replace infected cells that have been immunologically attacked. Alternatively, viral functions may play a direct role in mediating oncogenesis [4]. In Egypt, HBV and HCV are considered major health problems and disease prognosis may be worse in conjunction with schistosomiasis (Attia, 1998).

The development and progression of HCC are caused by the accumulation of genetic changes resulting in altered expression of cancer-related genes, such as oncogenes, tumor suppressor genes, and genes involved in different regulatory pathways [5,6]. Therefore, identification of new molecular parameters is important for cancer research and treatment. It is now possible to use profiling techniques such as cDNA array to identify genes that play important roles in human carcinogenesis [5]. Identification and monitoring of gene expression profile changes in HCC specimens will not only explain the cause(s) of pathological changes, but will also provide opportunity to identify novel targets for disease detection and intervention. In this study, we investigated the gene expression profile in Egyptian patients with HCV-associated HCC. We also evaluated the prognostic and predicative value of these genes and the possibility of defining candidate genes for molecular target therapy.

## Methods

### Patients

The study included 17 patients who attended the National Cancer Institute (NCI), Cairo University, and were consecutively diagnosed with HCC. The clinico-pathological features of the studied subjects are shown in table 1. Tumors and their adjacent non-neoplastic tissues together with venous blood samples were obtained from patients at the operation theatre. The study was conducted in compliance with the Helsinki Declaration and was approved by the senior staff committee and by a board regulating non-intervention study comparable to an institutional review board. All involved patients gave a written informed consent. Tissues were immediately cut into three parts; one piece was processed for routine histolopathological examination to confirm diagnosis, determine the pathological features of the tumor and assess tumor: normal ratio. The second and third portions were immediately snap-frozen and stored in liquid nitrogen for RNA and DNA extraction. Patients' profiles were extracted from the medical records.

**Table 1:** Clinical characteristic of the studies patients

**s.n.**	**Diagnosis**	**Grade**	**CAH**	**cirr.**	**AFP**	**HCV**	**HBV**
1	HCC	II	III	yes	14167	POS	NEG

2	HCC	III	III	yes	13.7	POS	NEG

3	HCC	II	No	yes	67	POS	NEG

4	HCC	II	No	no	32	POS	NEG

5	HCC	II	II	no	997	POS	NEG

6	HCC	II	III	yes	105.8	POS	NEG

7	HCC	II	III	yes	2322	POS	NEG

8	HCC	I	III	yes	63	POS	NEG

9	HCC	II	No	no	2.8	POS	NEG

10	HCC	III	No	Yes	1136	POS	NEG

11	HCC	II	No	No	107	POS	NEG

**12**	**HCC**	**II**	**No**	**No**	**137**	**POS**	**NEG**

**13**	**HCC**	**I**	**No**	**no**	**37.5**	**POS**	**NEG**

**14**	**HCC**	**II**	**III**	**Yes**	**60**	**POS**	**NEG**

**15**	**HCC**	**II**	**No**	**Yes**	**6.3**	**POS**	**NEG**

**16**	**HCC**	**III**	**III**	**Yes**	**600**	**POS**	**NEG**

**17**	**HCC**	**II**	***III***	**no**	**2.7**	**POS**	**NEG**

### Serological markers

Serological markers for HBV infection ([HBsAg and antibodies to hepatitis B core antigen [anti-HBc]) were detected with current standard assays (enzyme immunoassay [EIA]; Innogenetics, Belgium). A sample was considered HBV positive if it was positive for HBsAg or anti-HBc antibodies, or both. Antibodies to HCV were detected with HCV EIA version 3.0 (Innogenetics, Belgium). All serologic assays were done according to manufacturer's instructions.

### Detection of HCV-RNA

RNA was extracted from patients' sera according to manufacturer's instructions by Qiagen (Germany). The RT-PCR was performed as previously described by **Zekri et al. **[7].

### Detection of HBV DNA

DNA was extracted from frozen liver tissues of each patient according to the standard protocol of Mahoney (1996). All DNA extracts were analyzed for HBV genomes with three different polymerase chain reaction (PCR) assays to detect the *S, X *and core genes as previously described [8] to exclude occult HBV infection.

### cDNA Microarrays

RNA extraction from tissues: RNA was prepared from tumor samples and their adjacent non-neoplastic tissues. Each sample was tested in triplicate on array 15K (Array-I) supplied from Fox Chase Cancer Center . Briefly, RNA was extracted by homogenization (Polytron; Kinematica, Lucerne, Switzerland) in TRIzol reagent (Gibco BRL) at maximum speed for 90–120s. The homogenate was incubated for 5 min at room temperature. A 1:5 volume of chloroform was added, and the tube was vortexes and subjected to centrifugation at 12,000 g for 15 min. The aqueous phase was isolated, and one-half of the volume of isopropanol was added to precipitate the RNA. Purification was then performed with the Qiagen RNeasy Total RNA isolation kit according to manufacturer's specifications (Qiagen, Germany). The purified total RNA was finally eluted in 10 ul of diethyl pyrocarbonate-treated H2O, and the quantity and integrity were characterized using a UV spectrophotometer (Nanodrop). RNA was electrophoresed on an ethidium bromide stained agarose gel. It showed discrete bands of high molecular weight RNA between 7 Kb and 15 Kb, two predominant ribosomal RNA bands at approximately 5 Kb (28S) 2 Kb (18S), and low molecular weight RNA between 0.1 and 0.3 Kb (tRNA, 5S). The isolated RNA has an A 260/280 ratio of 1.9–2.1.

### RNA Labeling

Probes for microarray analysis were prepared from RNA templates by the synthesis of first strand cDNA containing amino-allyl-labeled nucleotides (Sigma Cat # A0410), followed by a covalent coupling labeled cDNA to the Cy Dye Ester to the NHSester of the appropriate Cyanine fluor, Cy3-ester (Amersham Pharmacia, Cat# PA23001) and Cy5-ester (Amersham Pharmacia, Cat# PA25001). This was followed by purification of the two probes by passing through a Microcon 30 columns (Millipore, Bedford, MA) according to the manufacturer's instructions.

### Hybridization

Hybridization occurred in 1× hybridization buffer containing 50% formamide, 5 × SSC, and 0.1% SDS. Prior to hybridization, the free amino groups on the slide were blocked or inactivated in the pre-hybridization solution containing 1% bovine serum albumin (BSA; Sigma Cat# A-9418), 5 × SSC and 0.1% SDS.

### Data Collection

Primary data from image files were obtained using Scan Array Express II (Perkin Elmer, USA), a confocal laser scanner capable of interrogating both the Cy3- and Cy5-labeled probes and producing separate images for each and then normalized using intensity and spatially dependent method, as previously described [9].

Following image processing, the data generated from the arrayed genes were further analyzed before differentially expressed genes could be identified. The first step in this process was the normalization of the relative fluorescence intensities in each of the two scanned channels. We calculated the normalization factors for each step of the experiment as follows: First, we used total measured fluorescence intensity in order to make the total mass of RNA labeled with either Cy3 or Cy5 equal. The total integrated intensity across all the spots in the array must be equal for both channels. Second, we used the scatter plot of Cy5/Cy3 of genes. (The scatter plot of Cy5/Cy3 of all genes is statistically examined). The scatter plots of the values of Cy3 and Cy5 fluorescent signals also revealed a pattern of distribution and were clustered in a diagonal line. A high correlation was observed in all samples and showed that there was a high reliability in the experiments by the cDNA microarray analysis of these samples. Third, we used some subsets of housekeeping genes that had already existed on each microarray chip. The ratio of measured Cy5 to Cy3 for these genes was modeled, and the mean of the ratio was adjusted to 1.

We applied the hierarchical clustering method to both genes and samples by using the Pearson r test as the measure of similarity and average linkage clustering as described previously [10]. To obtain reproducible clusters, we used only selected genes that passed the cut-off filter. The analysis was performed using a web-available software ("Cluster" and "Tree View") and confirmed by Genesis software, a tool that uses *k*-means clustering function and that was presented as a gift by Dr. Alexander Sturn, Graz University of Technology, Graz, Austria. We applied a hierarchical clustering algorithm to all of the selected genes. Information about genes participating in different function were obtained from Onto-Express Soft  as a gift. Information about genes participating in known signaling pathways was derived from Entrez Gene  and KEGG pathway  databases. To identify members of particular pathways, we combined the KEGG gene number with the identifier/accession number.

### Validation of the micrroarray results

#### PCR amplification of the studied genes

The genes selected from tables including 5 and 6 for the validation study were NOTCH4, VISA, CDK-7, ISGF3G, TNFα, and BAX genes (table 2) in which these genes were correlated to the cell cycle regulation pathways. The RT-PCR and quantification were performed in a 50 μl reaction volume. All samples were analyzed twice by the RT-PCR on different days with different RT-PCR mix to ensure reproducibility of results. Ten samples of human DNA and RNA were extracted from PBL and normal liver tissue used to optimize the best conditions for the multiplex PCR of *B-actin *gene versus each of the studied genes. Quantification of the studied genes were performed as previously described by Zekri et al. 2000

**Table 2:** Primer Sequences of the Studied Genes

**Gene name**	**Primer sequence**	**Annealing temp.**
B-actin	s:5'-ACA CTG TGC CCA ACG AGG-3' as:5'-AGG GGC CGG TCA TAC T-3'	55–59

NOTCH4	s:5'-GAG GAC AGC ATT GGT CTC AAG G-3' as:5'-CAA CTC CAT CCT CAT CAA CTT CTG-3'	60.4

VISA	s:5'-TGC CGT TTG CTG AAG ACA A-3' as:5'-TTC GTC CGC GAG ATC AAC T-3'	56.8

CDK-7	s:5'-CGG GCT TTA CGG CGC CGG ATG G-3' as:5'-CCC TCA GTA GTA AAA TGT TGT CC-3'	60

ISGF3G	s:5'-CTG GCA CAT GGC ACA CAC-3' as:5'-CAT CAA AGC GAC AGC ACA GT-3'	59

TNF-α	s:5'-ACA AGC CTG TAG CCC ATG TT-3' as:5'-AAA GTA GAC CTG CCC AGA CT-3'	57.9

BAX	s:5'-CAT GGA ACT GAT GAT GAT GAA-3' as:5'-CTC CAA CGA AAA ATG ATA-3'	60

### Statistical Analysis

The results were analyzed using Graph pad prism computer program (Graph pad software, San Diego, USA). For gene expression analysis, Mann Witney Test was used for numeric variables, and Chi square or Fisher's exact Test was used to analyze categorical variables. The p value was considered significant when P ≤ 0.05. We used Scan Array Express II (Perkin Elmer, USA) software for image processing. This software uses a threshold algorithm that separates spots from the background, allowing a grid to be laid across the spots. Having found a grid, spots are found within each grid element. The local background is calculated, and the background is subtracted. The integrated intensities were calculated for both the Cy3 and Cy5 channels. Measured intensities were analyzed using the Genesis software and R program that detect the up- and down-regulated genes according to the ratio in their software's.

## Results

In order to separate genes that are truly differentially expressed from those influenced by random changes, we conducted three independent microarray assays starting from independent mRNA isolations and defined differential expression based on their consensus. Three experiments were independently performed with each sample from the seventeen different HCC patients. When the triplicate experiments were compared, a percentage of reproducibility ranging from 67 to 90% was observed. Our results indicated a global reproducibility of results, with some discrepancies in the genes expressed. We therefore decided to perform the different analyses, taking into account all three experiments. Accordingly, 446 (Additional file 1) genes of known function were differentially expressed in our HCC cases out of the 15,645 studied genes.

Of the 446 genes, 180 showed up-regulation (additional file 1), 98 are involved in biological processes (table 3), 145 in molecular function (table 4), 98 in cellular components and seventeen are involved in 28 different pathways (table 5). The most frequent genes are protein phosphatase 3 (PPP3R1), which are involved in 10 different pathways, fibroblast growth factor receptor 1 (FGFR1), Cas-Br-M ecotropic retroviral transforming sequence b (CBLB) and spleen tyrosine kinase (SYK) in three pathways' and bone morphogenetic protein 8a (BMP8A), laminin alpha 3 (LAMA3), cell division cycle 23 (CDC23) in 2 pathways and NOTCH4 which regulate the Notch signaling pathway.

**Table 3:** Genes involved in the Biological Process of the up-regulated genes

Biological Process	Gene No.	Gene Name
Transcription	22	PWP1, ZNF202, SIN3A, CRSP3, SUD33, **CDK7**, **BCOR**, RNF2, ZNF77, **SND1**, **SOX30**, PHD finger protein 3, NOTCH4, CBX3, MNAT1, MAF, RXRA, TEAD3, SMAD6, MYC Binding protein 2 and SP11O

Regulation of transcription, DNA-dependent	17	**SMAD6**, CBX3, RNF2, SND1, **ARNT**, **SPEN**, TEAD3, PHF3, LOC643641, **MYCBP2**, SP110, SUDS3, ZNF77, ZNF202, **RXRA**, **MAF**

Biological process unknown	9	FETUB, UBQLN4, SURF5, PRCC, HSD17B1, ZNF77, BTBD1, SIPAILI, RXR4

Proteolysis	8	TMPRSS4, ADAMTS7, MMP23B, AZU1, PGM5P1, SPPL2B, ICEBERFG (caspase-1 inhibitor), GZMK

Protein amino acid phosphorylation	7	MAP4K4, CDK7, DYRK2, SYK, FGFR1, MARK4, RIPK2 (down regulate TLR 2/3/4, IL1 and IL8 receptor

Transcription from RNA polymerase II Promotor	6	TCEA1, TEAD3, MNAT1, SOX30, CRSP3

Intracellular signaling cascade	6	SH2B3, MCF2L, SYK, Rho GTPase activating protein, CAPS, CHN1

Ubiquity cycle	6	OTUB2, MYCBP2, CBLB, FBO31, CDC23, VPS8

Signal transduction	6	ARNT, RXRA, GAS6, TNFSF13B, ADORA1, RIPK2

Cell proliferation	6	CDK7, MNAT1, SYK, CSE1L, GAS6, TNFSF13B

Metabolism	5	QDPR, ATP2C1, HSD17B1, GSTM4, ECHS1

**Table 4:** Genes involved in the molecular function of the up regulated genes

**Molecular Fuction**	**Gene No.**	**Gene Name**
Protein Binding	28	MICA, SIPA1L1, SIN3A, VPS8, BTBD1, FBLN2, SUDS3, SYK, ARNT, RNF2, MYRIP, RIPK2, CBLB, SURF5, PHF3, NOTCH4, TNKS, LSM2(Bac-Bax), PSCD1, CBX3, MNAT1, RXPAP, FGFR1, ICEBERG(caspase-1-inhibitor), POP7, SMAD6, MYCBP2(MYC binding protein-2), SP110

Nucleotide binding	16	ABCG2, TNRC6B, SYK, MAP4K4, ATP6VIA, SPEN, MARK4, CUGBP2, CDK7, RAB14, MATR3, ATP2C1, FGFR1, RBM25, DYRK2, RIPK2

Metal ion binding	16	CHN1, RNF2, ADAMTS7, TCEA1, ATP6V1A, PLOD1, VPS8, PHF3, MYCBP2, MNAT1, MATR3, SP110, ZNF77, MYRIP, ZNF202, RXRA

Zinc ion binding	16	CHN1, RNF2, ADAMTS7, TCEA1, CBLB, VPS8, PHF3, MYCBP2, MNAT1, MATR3, SP110, ZNF77, MYRIP, ZNF202, MMP23B, RXPA

ATP binding	11	ABCG2, SKY, MAP4K4, ATP6V1A, MARK4, CDK4, DHX36, ATP2C1, FGFR1, DYRK2, RIPK2

Transferase activity	11	SYK, MAP4K4, HS2ST1, LYPLA3, MARK4, CDK7, FGFR1, DYRK2, RIPK2, GSTM4, TKT

Molecular Function unknown	10	WFDC1, FETUB, GPM6B, UBQLN4, PHF3, SURF5, PRCC, ZNF77, SIPA1L1, RXRA

Nucleic acid binding	9	EIF5A2, SND1, SPEN, POP7, LOC643641, MATR3, DHX36, RBM25, ZNF202

Calcium ion binding	9	GAS6, RCN1, CBLB, PPP3R1, FBLN2, ATP2C1, TKT, NOTCH4, CAPS

Hydrolase activity	7	LYPLA3, ITPA, TATDN2, POP7, DHX36, ATP6VIA, ATP2C1

DNA binding	6	SP110, TCEA1, SOX30, RNF2, ZNF77, SPEN

RNA binding	6	FNBP1, AKAP1, MATR3, LSM2, SPEN, CUGBP2

**Table 5:** Up regulated gene symbols and related pathway.

**Rank**	**Database Name**	**Pathway Name**	**Gene Symbole**
1	KEGG	Natural killer cell mediated cytotoxicity	PPP3R1, MICA, SYK,

2	KEGG	B cell receptor signaling pathway	PPP3R1, SYK

3	KEGG	TGF-beta signaling pathway	SMAD6, BMP8A

4	KEGG	T cell receptor signaling pathway	PPP3R1, CBLB

5	KEGG	Cell cycle	CDC33, CDK7

6	KEGG	MAPK signaling pathway	TGFR1, PPP3R1, MAP4K4

7	KEGG	Ubiquitin mediated proteolysis	CDC23

8	KEGG	Notch signaling pathway	NOTCH4

9	KEGG	Hedgehog signaling pathway	BMP8A

10	KEGG	Long-term potentiation	PPP3R1

11	KEGG	Adipocytokine signaling pathway	RXRA

12	KEGG	Complement and coagulation cascades	SERPINA5

13	KEGG	VEGF signaling pathway	PPP3R1

14	KEGG	Fc epsilon RI signaling pathway	SYK

15	KEGG	Adherens junction	FGFR1

16	KEGG	Apoptosis	PPP3R1

17	KEGG	Antigen processing and presentation	TAPBP

18	KEGG	ECM-receptor interaction	LAMA3

19	KEGG	Tight junction	EPB41L2

20	KEGG	Axon guidance	PPP3R1

21	KEGG	Insulin signaling pathway	CBLB

22	KEGG	Wnt signaling pathway	PPP3R1

23	KEGG	Jak-STAT signaling pathway	CBLB

24	KEGG	Calcium signaling pathway	PPP3R1

25	KEGG	Focal adhesion	LAMA3

26	KEGG	Regulation of actin cytoskeleton	FGFR1

27	KEGG	Cytokine-cytokine receptor interaction	TNFSF13B

28	KEGG	Neuroactive ligand-receptor interaction	ADORA1

Down-regulation of 134 genes was reported (additional file 1). 44 genes are involved in biological processes (table 6). The most characteristic genes were related to virus response like virus-induced signaling adapter (VISA), interferon-stimulated transcription factor 3 (ISGF3G), Gardner-Rasheed feline sarcoma viral (v-fgr) oncogene homolog (FGR). Eighty-three genes are involved in molecular function (table 7) mainly alpha-2 macroglobulin and protease serine12 (RPSS12), 60 genes in cellular component and 25 are involved in 20 different pathways (table 8). The most frequent one is the integrin alpha V alpha polypeptide antigen CD51 (ITGVA) in 4 pathways followed by lymphotoxin alpha (TNF superfamily, member 1) (LTA) in 3 pathways and alpha-2-macroglobulin (A2M), phosphorylase kinase alpha 2-liver (PHKA2) and MAGI1 membrane associated guanylate kinase 1 (MAGI1) in 2 pathways.

**Table 6:** Genes involved in the Biological Process of the down regulated genes

Biological Process	Gene No.	Gene Name
Transcription	6	HSF4, ISGF3G, ZNF644, AVIAN, KLF6, SP4 transcription factor

Biological process unknown	6	ARPP-21, KLF6, NAT10, SLTT2, GSTA2, GLTSCR2

Regulation of transcription, DNA-dependent	6	HSF4, ZNF644, ISGF3G, VENTX, ISGF3G, AVIAN

Immune response	4	ISGF3G, AVIAN, TNF (superfamily, member1), OAS2

Protein transport	4	VPS33B1, SEC61A1, TOMM40, VPS35

Apoptosis	3	RNF34, ATG5, RAD21

Ubiquitin cycle	3	FBXO22, RNF34, USP30

Carbohydrate metabolism	3	PHKA2, MGAT4B, TALDO1

Transcription from RNA polymerase II Promotor	3	ISGF3G, PTTG1, AVIAN

Cell adhesion	3	NRXN2, MAGI1I, TGAV

Response to virus	3	VISA, ISGF3G, FGR

**Table 7:** Genes involved in the molecular function of the down regulated genes

**Molecular Function**	**Gene No.**	**Gene Name**
Protein Binding	14	PRPF6, SPINK1, RAD21, NRXN2, RNF34, CORO2A, TALD01, PTTG1, NAGK, VPS35, ISGF3G, TTGAV, HOMER3, DMXL1,

Transferase activity	9	NDST2, GSTA2, OAS2, FGR, TALDO1, NAGK, FTCD, NAT10, MAGI1,

Metal ion binding	9	ZSWIM1, THAP4, RNF34, ZNF644, ZC3H7B, ISGF3G, KLF6, IMPDH2, SP4

Zinc ion binding	8	ZC3H7B, ZNF644, KLF6, ISGF3G, RNF34, THAP4, SP4, ZSWIM1

Nucleotide binding	8	NAT10, MYH8, MAGI1, NAGK, APRL1, FGR, A2BP1, CUGBP2

Molecular Function unknown	8	ARPP-21, C9ORF156, KLF6, NAT10, PTTG1, TMEPA1, GLSCR2, TOMM40

Calcium ion binding	7	CANX, EFCAB2, FGEQ, THBD, ITGAV, FGG, SLIT2

ATP binding	7	NAT10, MGC16169, MYH8, MAGI1, NAGK, FGR, OAS2

Transcription factor activity	5	HSF4, VENTX, ISGF3G, PTTG1, AVIAN

Nucleic acid binding	4	ZC3H7B, ARPP-21, KLF6, A2BP1

DNA binding	4	ZNF644, KLF6, THAP4, SP4

Other important genes interleukin-8 binding protein, alph-2 macroglobulin and RPSSS12

**Table 8:** Down regulated gene symbols and related pathway.

**Rank**	**Pathway Name**	**Gene Symbol**
1	Complement and coagulation cascades	FGG, THBD, A2M, SERPINA5

2	Antigen processing and presentation	LTA, CANX

3	Dentatorubropallidoluysian atrophy (DRPLA)	MAGI1

4	Amyotrophic lateral sclerosis (ALS)	SLC1A2

5	Alzheimer's disease	A2M

6	Cell adhesion molecules (CAMs)	NRXN2, ITGAV

7	Regulation of autophagy	ATG5

8	SNARE interactions in vesicular transport	C1orf142

9	Type I diabetes mellitus	LTA

10	ECM-receptor interaction	ITGAV

11	Cell cycle	PTTTG1

12	Tight junction	MAGI1

13	Axon guidance	SLIT2

14	Insulin signaling pathway	PHKA2

15	Jak-STAT signaling pathway	ISGF3G

16	Calcium signaling pathway	PHKA2

17	Focal adhesion	ITGAV

18	Regulation of actin cytoskeleton	ITGAV

19	Cytokine-cytokine receptor interaction	LTA

20	MAPK signaling pathway	MAPK8IP3

**Figure 1:**
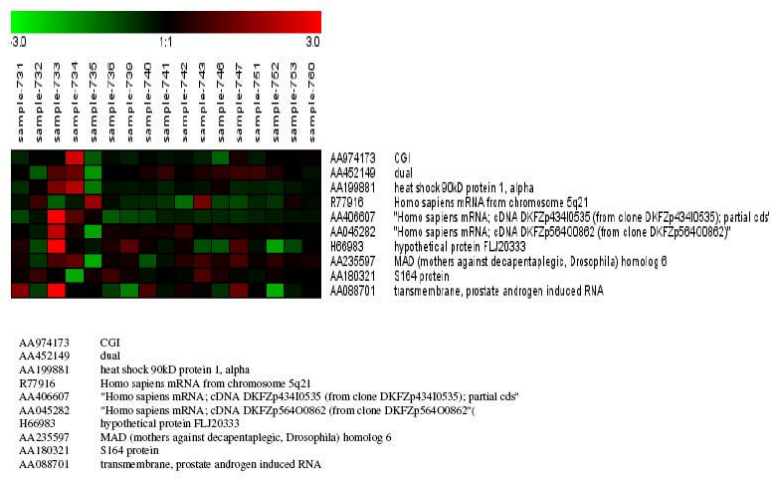
Differentially expressed genes in HCC which show a significant difference between high and low AFP.

**Figure 2:**
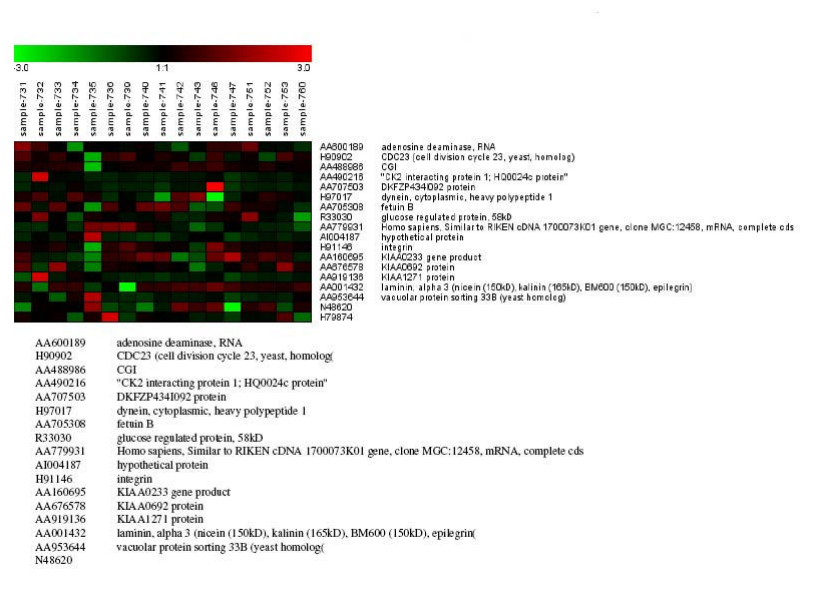
Differentially expressed genes in HCC which show a significant difference between presences of either cirrhosis or CAH.

**Figure 3:**
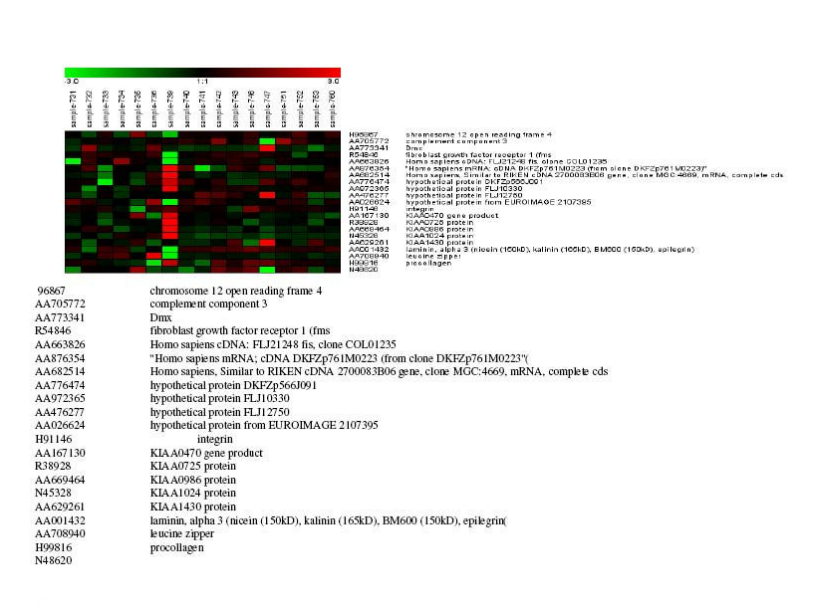
Differentially expressed genes in HCC which show a significant difference between presences of HCV with cirrhosis in array I.

Out of these 446 genes, which were differentially expressed, only 10 showed significant differences between cases with high (≥ 600 IU/ml) and low (≤ 200 IU/ml) AFP levels (figure 1). One of them is SMAD6, which is involved in the FGF-β signaling pathway. Eighteen genes, including CDC3 and LAMA3 which are involved in the same pathways, showed significant differences between HCC cases with either cirrhosis or chronic active hepatitis (figure 2), whereas 21 genes, including FGFR1 and LAMA 3, showed significant differences between HCC with cirrhosis and HCC without cirrhosis. This is considered a unique phenomenon in Egyptian HCC patients (figure 3). FGFR1 is involved in adherense junction, regulation of actin cytoskeleton and MAPK, whereas LAMA 3 is involved in ECM receptor interaction and focal adhesion. Using this approach, we identified many genes, which are potentially implicated in HCC. These genes were not previously identified by conventional cDNA microarray assays (see additional file 1).

Confirmation of the array results was performed by RT-PCR in which limited studies were done on these genes and the Bax gene was chosen from our previous published data (not included). The relative value for each gene was calculated in relation to normal pooled liver tissues level. Then we classified cases according to the level of expression into those with reduced expression and those with over-expression for each gene and patient. Accordingly, genes were classified into two groups: group I included genes showing reduced expression [*ISGF3G *(76%), *TNF*-α(88%), *VISA *(82%)], and group II included genes showing over-expression [CDK7 (70%), NOTCH4, (64%,), BAX (78%)] (table 9).

**Table 9:** The correlation between the expression level of the studied genes and clinicopathological features of hepatocellular carcinoma cases

**Reduced expression**				**Over expression**		
Variable	ISGF3G	TNF-α	VISA*	CDK-7	NOTCH4	BAX
Total: 17 (%)	13 (76)	15 (88)	14 (82)	12 (70)	11 (64)	13 (76)

Age (Mean ± SD) (57 ± 10.2)						
< 57 = 10(59)	8(80)	9(90)	8(80)	7(70)	7 (70)	8(80)
> 57 = 7(41)	5(71)	6(85)	6(85)	5(71)	4 (57)	5(71)

Gender						
Male:13(76)	10(77)	12(92)	11(85)	10(77)	9(69)	11(85)
Female:4(24)	3(75)	3(75)	3(75)	2(50)	2(50)	2(50)

Tumor site						
Rirht = 11(65)	9(82)	10(90)	8(73)	7(64)	6(55)	9(82)
Left = 6(35)	4(66)	5(83)	6(100)	5(83)	5(83)	4(66)

Tumor size						
≤ 8 = 12(70)	10(83)	10(83)	11(91)	9(75)	8(66)	9 (75)
> 8 = 5(30)	3(60)	5(100)	3(60)	3(60)	5(100)	4 (80)

Grade						
I+II = 14(82)	10(71)	13(93)	12(85)	10(71)	8(57)	10(71)
III = 3(18)	3(100)	2(66)	2(66)	2(66)	3(100)	3(100)

Safety margin						
Pos 4 (24)	2(50)	3(75)	4(100)	3(75)	3(75)	3(75)
Neg 13(76)	11(85)	12(92)	10(77)	9(69)	8(61)	10(77)

Invasion						
Pos 9 (53)	7(77)	8(88)	8(88)	8(88)	9(100)	6(66)
Neg 8(47)	6(75)	7(87)	6(75)	4(50)	2(25)	7(87)

Cirrhosis						
Present: 10(59)	8(80)	9(90)	8(80)	7(70)	7(70)	9(90)
Absent: 7(41)	5(71)	6(85)	6(85)	5(71)	4(57)	4(57)

CAH						
Pos: 9 (53)	7(77)	8(88)	7(77)	6(66)	6(66)	8(88)
Neg: 8(47)	6(75)	7(87)	7(87)	6(75)	5(63)	5(63)

*VISA (also known as MAVS, IPS-I or CARDIF)

## Discussion

The rising trend of HCC incidence has been associated with increased prevalence of HCV infection though the fundamental mechanism(s) by which HCV is related to HCC is (are) not definitely known [11]. Recent progress in molecular biology has improved our understanding of the genesis of a wide range of human neoplasms. In HCC, several groups have reported microarray-based profiling data and illustrated that genes with altered expression in most of HCCs may serve as molecular diagnostic markers and candidates of HCC-therapeutic targets or may play causal roles in hepatocarcinogenesis.

In this study, out of the 17 samples with HCV-associated HCCs and their adjacent normal tissues, 446 genes of known function were differentially expressed out of the 15,645 studied genes. Out of these, 180 showed up-regulation and 134 showed down-regulation.

We compared the HCC up- and down-regulated genes identified by the cDNA microarray assays in accordance with their potential molecular functions, implicated in biological processes and sub-cellular localization. Up-regulated genes had a strong association with the regulation of cell cycle progression, transcription, nucleic acid metabolism, and protein metabolism. The HCC down-regulated genes, on the other hand, tended to be related to the loss of the normal physiological function of the hepatocytes. They also tended to be related to the impairment in cellular defense activities, in maintaining cell ion homeostasis and in cellular responses to extrinsic stresses, including wounding and external biotic stimuli. In addition, genes related to cell responses to external growth stimuli and signal transduction and related to cell morphogenesis and biogenesis were more frequently found in the HCC up-regulated genes. On the other hand, the HCC up-regulated genes had more genes with their products distributed in cell nucleus, while the HCC down-regulated genes had more genes of secreted proteins.

Among the up-regulated genes, we focused on LAMA3 as a possible molecular target for HCC therapy. Ln-5 is a heterotrimeric glycosylated protein formed by α3, β3, γ2 chains assembled with disulfide bonds that are the product of three different genes (LAMA3, LAMB3, and LAMC2), respectively [12]. It is a main component of the BM structure, where it promotes different functions such as adhesion or migration. Therefore, a number of reports suggest its involvement in the spread and metastasis of cancer cells [[13-15] and [16]]. Ln-5 is widely expressed in the human body; however, no study has yet investigated the expression of Ln-5 in the liver under pathological conditions such as HCC or the expression of all of the chains in cancer tissues in the same patient.

Our study showed up regulation of protein phosphatase 3 receptor gene (PP3R) in most of the HCC cases. To our knowledge, this is the first time to detect this gene in the HCC cases. PPP3 (formerly PP2B, Calcineurin) is a serine/threonine protein phosphatase. Some studies showed that the PPP3RL gene is localized on human chromosome 9q22, and transcripts of PPP3RL gene are specifically expressed in the testis [17].

Transforming growth factor-b1 (TGF-b1) is an important growth regulatory molecule that triggers apoptosis in hepatocytes [18]. HCC is resistant to TGF-b1 even though the latter is transcriptionally up-regulated in tumor cells and is commonly elevated in the sera of HCC patients [19,20]. TGF-b1 may thus promote tumor growth, in part by killing or inhibiting the growth of surrounding hepatocytes.

Some data suggest that the resistance of HCC to TGF-b1 is associated with mutation and loss of the receptor that mediates TGF-b1 signaling [21], although this has not been consistently observed [22]. Alternatively, HBxAg may suppress the expression of TGF-b1 type II receptor [23] or up-regulates the TGF-b1 gene by HBxAg [24], probably by the constitutive activation of transcriptional complexes containing Smad4 [25], which mediates TGF-b1 signaling. TGF-b1 signaling also promotes the development of fibrosis and cirrhosis in patients with chronic liver disease (CLD) [26]. Some studies showed an increased expression of TGFa, aFGF and HGF and their respective receptors during hepatocarcinogenesis [27]. Among them, TGFa and aFGF appear to be the major growth factors produced by tumor cells and may therefore be the main contributors to the progression of HCC. This was also reported in our study.

Similarly, Ogasawara et al., 1996; Tsou et al., 1998 have reported over-expression of the fibroblast growth factor receptor (FGFR1) in HCC in relation to the proliferation of cancerous cells. FGF has been considered to contribute to various human tumors and malignant growth of neoplasm. Hepatocellular carcinoma (HCC) is a typical hypervascular tumor, and it is suggested that FGF may be involved in hepatocarcinogenesis [28,29].

The spleen tyrosine kinase (SYK) is a tumor/metastasis suppressor gene recently found to be silenced through DNA methylation in breast cancer and T-lineage acute lymphoblastic leukemia. Loss of SYK expression has been implicated in increased invasiveness and proliferation of breast tumors [30]. Our data regarding the expression of SYK agree with the few available reports in this context where methylation andloss of SYK expression in HCC neoplastic tissues were found to be independent biomarkers of poor patient outcome [31].

Notch1 signaling may participate in the development of HCC cells by affecting multiple pathways that control both cell proliferation and apoptosis. In this study, we record up-regulation of NOTCH4 gene, which regulates Notch signaling pathway. This has also been reported by Runzi et al. 2003. Notch1 signaling-induced growth suppression is at least partially due to G0/G1 cell cycle arrest. Up-regulation of p53 expression and down-regulation of Bcl-2 may be related to Notch1 signaling-induced apoptosis. Therefore, Notch1 signaling can inhibit HCC growth through the induction of the cell cycle arrest and apoptosis [32].

IFN-β promoter stimulator (IPS)-1, also known as mitochondrial antiviral signaling protein (MAVS), virus-induced signaling adaptor (VISA), and CARD adaptor inducing IFN-β (Cardif), was recently identified as an adaptor linking RIG-I and Mda5 to the downstream signaling molecules. IPS-1 contains the CARD-like domain that is responsible for the interaction with that of RIG-I and Mda5. In addition, IPS-1 contains a transmembrane region that targets this protein to the mitochondrial outer membrane. IPS-1-deficient mice showed severe defects in both RIG-I- and Mda5-mediated induction of type I interferon and inflammatory cytokines and were susceptible to RNA virus infection. RNA virus-induced interferon regulatory factor-3 and nuclear factor *KB *activation was also impaired in IPS-1-deficient cells. IPS-1, however, was not essential for the responses to either a DNA virus or a double-stranded B-DNA. Thus, IPS-1 is the sole adapter in both RIG-I and Mda5 signaling that mediates effective responses against a variety of RNA viruses. The Virus-induced signaling adaptor (VISA) is essential for host innate immune responses against double-stranded RNA viral infection and viral replication. Down-regulation of this gene in our HCC cases may be related to the replication of the hepatitis virus. It is an adaptor that activates the transcription of the nuclear factor κB (NF-κB) and interferon regulatory factor 3 (IRF3), which regulate the expression of type I interferon.

The expression of IL-8 in human HCC has more relevance to metastasis than to angiogenesis or cell proliferation. The expression of IL-8 did not significantly correlate with micro-vessel count in HCC tissues, but the incidence of microscopic vessel invasion was significantly higher in IL-8-positive than in IL-8-negative tissues. In this study, we found decreases in the expression of IL-8 binding protein which leads to the increase in the expression of IL-8. More IL-8 was expressed in HCCs at pathologic stage III/IV than in those at stage I/II [33].

A novel human malignancy-associated gene (MAG) expressed in various malignant tumors including glioblastomas and HCCs and in tumor pre-existing conditions such as hepatitis C virus- and hepatitis B virus-induced liver cirrhosis. This novel gene may play a role in the progression of premalignant conditions and in the development of HCC and other cancers [34].

The twenty-one genes, which showed significant differences between HCV-associated HCC with and without cirrhosis (figure 2 and 3), reveal a unique phenomenon in Egyptian cases and provide new focal points for cancer research.

We conclude that the up-regulated genes identified through the studied expression profiles of Egyptian HCC may shed light on the mechanisms of hepatic carcinogenesis.

To confirm our results, we checked several tumor-expressing genes to elucidate whether the identified genes correlate with previous published literature and to define a logical relation between the genes and hepatocarcinogenesis. We identified numerous genes that have previously been associated with liver carcinoma or other types of cancer [35,36].

## Competing interests

The authors declare that they have no competing interests.

## Authors' contributions

A-RNZ conceived of the study, carried out the microarray studies, participated in its design and coordination, Data analysis, drafted the manuscript and coordinate the whole work team. MMH was involved in sample collection, carried out the microarray studies, participated in the drafted the manuscript and performed the statistical analysis. AAB carried out the histolopathological examination. ZKH was involved in sample collection, carried out the microarray studies and editing the manuscript. MHK was responsible for the patient treatment and clinical data collection. TM coordinated the research effort. All six co-authors read and approved the final manuscript

## Supplementary Material

Additional file 1 List of the differentially express 446 gene out of the studied 15,400 gene studied by array-I. Click here for file
