# Supplementary material for: Genetic profile of Egyptian hepatocellular-carcinoma associated with hepatitis C virus Genotype 4 by 15 K cDNA microarray: Preliminary study
Source: BMC Res Notes. 2008 Oct 29;1:106. doi: 10.1186/1756-0500-1-106 (PMC2584108; doi:10.1186/1756-0500-1-106)
Supplement: Additional file 1 — List of the differentially express 446 gene out of the studied 15,400 gene studied by array-I. [file 1756-0500-1-106-S1.doc]

List of the differentially express 446 gene out of the studied 15,400 gene studied by array-I.

| 1. AA626878 | hypothetical protein FLJ22477 |
| --- | --- |
| 1. AA858390 | calcyphosine |
| 1. N34827 | glucuronidase, beta |
| 1. AA718954 | chromosome 9 open reading frame 11 |
| 1. AA701489 | Homo sapiens cDNA: FLJ21429 fis, clone COL04205 |
| 1. AA626281 | Homo sapiens clone CDABP0008 mRNA sequence |
| 1. AA018114 | Homo sapiens clone 24649 mRNA sequence |
| 1. AA018907 | protein phosphatase 3 (formerly 2B), regulatory subunit B (19kD), alpha isoform (calcineurin B, type I) |
| 1. AA625850 | butyrate |
| 1. AI016677 | Homo sapiens clone PP1744 unknown mRNA |
| 1. T62627 | interferon |
| 1. AA776327 | hypothetical protein |
| 1. AA670359 | Homo sapiens mRNA; cDNA DKFZp564B1162 (from clone DKFZp564B1162); complete cds |
| 1. AA083385 | BTB (POZ) domain containing 1 |
| 1. T67105 | G protein |
| 1. AA629603 | PTPL1 |
| 1. AA778874 | hypothetical protein FLJ11220 |
| 1. AA192422 | fucosyltransferase 8 (alpha (1,6) fucosyltransferase) |
| 1. AA598572 | spleen tyrosine kinase |
| 1. W16832 | C3H |
| 1. AA857804 | homolog of Xenopus Claspin |
| 1. N48891 | Stargardt disease 3 (autosomal dominant) |
| 1. AA668520 | asparaginyl |
| 1. AA873427 | Homo sapiens mRNA; cDNA DKFZp434M0420 (from clone DKFZp434M0420) |
| 1. R96235 | prothymosin, alpha (gene sequence 28) |
| 1. T90980 | splicing factor, arginine/serine |
| 1. H64973 |  |
| 1. AI024401 | DKFZP434J046 protein |
| 1. AA886870 | hypothetical protein FLJ11200 |
| 1. AA663589 | Homo sapiens HSPC302 mRNA, partial cds |
| 1. AA291389 | interferon |
| 1. AA865924 | hypothetical protein FLJ20464 |
| 1. N73548 | olfactory receptor, family 2, subfamily I, member 6 |
| 1. T67558 | androgen induced protein |
| 1. T84491 | CUG triplet repeat, RNA |
| 1. AI017640 | Homo sapiens mRNA; cDNA DKFZp434H1235 (from clone DKFZp434H1235); partial cds |
| 1. AA279755 | CD69 antigen (p60, early T |
| 1. N53411 | hypothetical protein DKFZp761O0113 |
| 1. R38360 | hypothetical protein |
| 1. AA504113 | M |
| 1. AA955007 | transaldolase 1 |
| 1. AA481503 | calmodulin 2 (phosphorylase kinase, delta) |
| 1. AA430032 | pituitary tumor |
| 1. N46452 |  |
| 1. AA876354 | Homo sapiens mRNA; cDNA DKFZp761M0223 (from clone DKFZp761M0223) |
| 1. R22625 | cyclin |
| 1. AA776474 | hypothetical protein DKFZp566J091 |
| 1. AA437126 | hypothetical protein AF053356_CDS3 |
| 1. AA707081 | Homo sapiens cDNA: FLJ23546 fis, clone LNG08361 |
| 1. AA976544 | hypothetical protein MGC2771 |
| 1. AA972365 | hypothetical protein FLJ10330 |
| 1. AA167130 | KIAA0470 gene product |
| 1. AA682514 | Homo sapiens, Similar to RIKEN cDNA 2700083B06 gene, clone MGC:4669, mRNA, complete cds |
| 1. R38928 | KIAA0725 protein |
| 1. AA669464 | KIAA0986 protein |
| 1. N45328 | KIAA1024 protein |
| 1. AA055350 | adenosine A2b receptor |
| 1. N26536 | ATPase, Cu++ transporting, beta polypeptide (Wilson disease) |
| 1. R76529 | R76247 | Homo sapiens clone 23568, 23621, 23795, 23873 and 23874 mRNA sequences |
| 1. AA927544 | KIAA0540 protein |
| 1. AA917649 | exosome component Rrp46 |
| 1. AA160692 | Homo sapiens mRNA for FLJ00116 protein, partial cds |
| 1. AA070501 | TBP |
| 1. W78011 | hypothetical protein FLJ10618 |
| 1. N68256 | lipase, hepatic |
| 1. AA454625 | hypothetical protein MGC5521 |
| 1. AA457216 | RAB22A, member RAS oncogene family |
| 1. AA477298 | branched chain keto acid dehydrogenase E1, alpha polypeptide (maple syrup urine disease) |
| 1. H24708 | discs, large (Drosophila) homolog 1 |
| 1. AA630633 | hypothetical protein FLJ12816 |
| 1. N69661 | uncharacterized hypothalamus protein HTMP |
| 1. H54286 | KIAA0561 protein |
| 1. AA883597 | nudix (nucleoside diphosphate linked moiety X) |
| 1. AA911236 | v |
| 1. AI018381 | twisted gastrulation |
| 1. AA043118 | chromosome 9 open reading frame 9 |
| 1. AA701963 | aldo |
| 1. AA598531 | ferrochelatase (protoporphyria) |
| 1. AA478158 | KIAA0494 gene product |
| 1. AA708793 | Homo sapiens mRNA for P53TG1 |
| 1. AA779931 | Homo sapiens, Similar to RIKEN cDNA 1700073K01 gene, clone MGC:12458, mRNA, complete cds |
| 1. N71435 | KIAA0701 protein |
| 1. AA704985 | phosphorylase kinase, alpha 2 (liver) |
| 1. AA699859 | Homo sapiens genomic DNA, chromosome 21q, section 4/105 |
| 1. N74593 | hypothetical protein FLJ11848 |
| 1. AA683351 | suppressor of white apricot homolog 2 |
| 1. AA683102 | RAD21 (S. pombe) homolog |
| 1. T95805 | putative mitochondrial outer membrane protein import receptor |
| 1. AA134771 | zinc finger protein 24 (KOX 17) |
| 1. AA905362 | ADP |
| 1. AA074614 | Homo sapiens, Similar to RIKEN cDNA 2410018G23 gene, clone MGC:11075, mRNA, complete cds |
| 1. H79874 |  |
| 1. AA626255 | enoyl Coenzyme A hydratase, short chain, 1, mitochondrial |
| 1. AA452840 | fibulin 2 |
| 1. AA884755 | FGFR1 oncogene partner |
| 1. AA704729 | Cas |
| 1. AA481759 | menage a trois 1 (CAK assembly factor) |
| 1. AA620894 | heterochromatin |
| 1. AA969787 | DKFZP434F091 protein |
| 1. N80764 | Homo sapiens cDNA: FLJ23494 fis, clone LNG01885 |
| 1. R22308 | nuclear phosphoprotein similar to S. cerevisiae PWP1 |
| 1. H99816 | procollagen |
| 1. AA952897 | nuclear factor of kappa light polypeptide gene enhancer in B |
| 1. AI015686 | glioma tumor suppressor candidate region gene 2 |
| 1. AA426086 | inactivation escape 2 |
| 1. AI004349 |  |
| 1. AA918907 |  |
| 1. AA428321 | N |
| 1. AA404609 | hypothetical protein FLJ22418 |
| 1. H41237 | KIAA0921 protein |
| 1. AA905273 | nectin 3; DKFZP566B0846 protein |
| 1. AA496947 | thymine |
| 1. AA505111 | copine III |
| 1. AA486228 | CGI |
| 1. H45303 | Homo sapiens clone CDABP0081 mRNA sequence |
| 1. AA984314 | BM88 antigen |
| 1. AA504160 | ATPase, H+ transporting, lysosomal (vacuolar proton pump), alpha polypeptide, 70kD, isoform 1 |
| 1. H61825 | Homo sapiens spinster |
| 1. AA609872 | hypothetical protein FLJ12553 |
| 1. AA707484 | Homo sapiens mRNA; cDNA DKFZp434A1014 (from clone DKFZp434A1014); partial cds |
| 1. AA629356 |  |
| 1. N55484 | aryl hydrocarbon receptor nuclear translocator |
| 1. AA488180 | papillary renal cell carcinoma (translocation |
| 1. AA193116 | glycerol |
| 1. H73640 |  |
| 1. AA905896 | melanoma antigen, family A, 2 |
| 1. AA425650 | thyroid hormone receptor interactor 8 |
| 1. H97017 | dynein, cytoplasmic, heavy polypeptide 1 |
| 1. AA862966 |  |
| 1. W72329 | lymphotoxin alpha (TNF superfamily, member 1) |
| 1. AA459006 | Homo sapiens cDNA: FLJ22063 fis, clone HEP10326 |
| 1. AA199881 | heat shock 90kD protein 1, alpha |
| 1. R72244 | 2' |
| 1. AA894634 | slit (Drosophila) homolog 2 |
| 1. AA918380 | diacylglycerol O |
| 1. T73468 | glutathione S |
| 1. AA772989 | Sp4 transcription factor |
| 1. AA873159 | apolipoprotein C |
| 1. H85094 | nucleosome assembly protein 1 |
| 1. T97303 | signal sequence receptor, alpha (translocon |
| 1. AA679000 | Splicing factor, arginine/serine |
| 1. AA719156 | testes |
| 1. N68172 | CGI |
| 1. AA864204 | x 001 protein |
| 1. AA670383 | KIAA0551 protein |
| 1. AA406607 | Homo sapiens mRNA; cDNA DKFZp434I0535 (from clone DKFZp434I0535); partial cds |
| 1. AA453813 | sialyltransferase 4C (beta |
| 1. H66983 | hypothetical protein FLJ20333 |
| 1. AA088701 | transmembrane, prostate androgen induced RNA |
| 1. AA045282 | Homo sapiens mRNA; cDNA DKFZp564O0862 (from clone DKFZp564O0862) |
| 1. R10382 | serine (or cysteine) proteinase inhibitor, clade A (alpha |
| 1. AA098892 | Homo sapiens, clone IMAGE:2958115, mRNA, partial cds |
| 1. AA777043 |  |
| 1. AA865224 | core promoter element binding protein |
| 1. AA999776 | heat shock transcription factor 4 |
| 1. AA962376 | HSPC182 protein |
| 1. AA883397 | hypothetical protein DKFZp762O076 |
| 1. T94279 | fibrinogen, gamma polypeptide |
| 1. AA278296 | mitogen |
| 1. AA856703 | vacuolar protein sorting 35 (yeast homolog) |
| 1. AA635186 | IMP (inosine monophosphate) dehydrogenase 2 |
| 1. R88535 | ataxin 2 |
| 1. AA775447 | alpha |
| 1. N71049 | serine (or cysteine) proteinase inhibitor, clade A (alpha |
| 1. H59861 | thrombomodulin |
| 1. AA918755 | frequenin (Drosophila) homolog |
| 1. AA780740 |  |
| 1. AA206614 | hypothetical protein FLJ11175 |
| 1. AA029934 | integrin, alpha V (vitronectin receptor, alpha polypeptide, antigen CD51) |
| 1. AA845156 | serine protease inhibitor, Kazal type 1 |
| 1. AA490216 | CK2 interacting protein 1; HQ0024c protein |
| 1. AA867983 | Homo sapiens mRNA for KIAA1764 protein, partial cds |
| 1. AA256172 | Gardner |
| 1. AA919136 | KIAA1271 protein |
| 1. AA775980 | solute carrier family 1 (glial high affinity glutamate transporter), member 2 |
| 1. N/A |  |
| 1. AA907721 | Homo sapiens cDNA: FLJ22097 fis, clone HEP17013 |
| 1. AA629022 |  |
| 1. AA705772 | complement component 3 |
| 1. N48620 |  |
| 1. AI004779 | DKFZP434A236 protein |
| 1. AA863296 | Homo sapiens cDNA: FLJ22448 fis, clone HRC09541 |
| 1. AA707503 | DKFZP434I092 protein |
| 1. AA676756 | KIAA1031 protein |
| 1. AA919115 | GTPase Rab14 |
| 1. AA865147 | KIAA1108 protein |
| 1. AA610004 | putative oncogene protein hlc14 |
| 1. AA918089 | Homo sapiens, clone IMAGE:3629896, mRNA, partial cds |
| 1. AA625899 | hypothetical protein FLJ14351 |
| 1. AA670270 | KiSS |
| 1. AA999953 | transmembrane protease, serine 4 |
| 1. H70866 | cathepsin O |
| 1. AA009629 | CGI |
| 1. AA521423 | deleted in cancer 1; RNA helicase HDB/DICE1 |
| 1. AA885476 | hypothetical protein FLJ20618 |
| 1. AA872274 | mannosyl (alpha |
| 1. AI004181 | Homo sapiens ubiquitin protein ligase (UBE3B) mRNA, partial cds |
| 1. AA682850 | hypothetical protein FLJ22609 |
| 1. AI017072 | myoneurin |
| 1. AA936779 | keratin 6A |
| 1. AA151428 | matrix metalloproteinase 23B |
| 1. H21045 | adenosine A1 receptor |
| 1. AA902808 | tankyrase, TRF1 |
| 1. AA913804 | receptor |
| 1. AA150491 | WAP four |
| 1. AA999976 | glutathione S |
| 1. AA644559 | LIM domain only 4 |
| 1. AA099360 | hypothetical protein FLJ20285 |
| 1. AA045458 | M |
| 1. AA677246 |  |
| 1. N47469 | zinc finger protein 281 |
| 1. AA486570 | glutathione S |
| 1. AA461110 | growth arrest |
| 1. AA173423 | hypothetical protein |
| 1. W49494 | KIAA0375 gene product |
| 1. AA774824 | Homo sapiens clone 23649 and 23755 unknown mRNA, partial cds |
| 1. AA160484 | Homo sapiens PAC clone RP5 |
| 1. AA625853 | Homo sapiens MARKL1 mRNA for MAP/microtubule affinity |
| 1. R33219 | hypothetical protein DKFZp547A023 |
| 1. AI017231 | GTT1 protein |
| 1. AA464852 | Notch (Drosophila) homolog 4 |
| 1. AA457719 | reticulocalbin 1, EF |
| 1. R07846 | Homo sapiens mRNA full length insert cDNA clone EUROIMAGE 208948 |
| 1. AA421286 | host cell factor 2 |
| 1. R33852 | syntaxin 11 |
| 1. R89358 | aldo |
| 1. H96867 | chromosome 12 open reading frame 4 |
| 1. AA862693 | KIAA0804 protein |
| 1. R53891 | CGI |
| 1. AA001432 | laminin, alpha 3 (nicein (150kD), kalinin (165kD), BM600 (150kD), epilegrin) |
| 1. R42852 | glycoprotein M6B |
| 1. AA883675 | ATPase, Ca++ transporting, type 2C, member 1 |
| 1. H28922 | MCF.2 cell line derived transforming sequence |
| 1. R63802 | ring finger protein 2 |
| 1. R37938 | KIAA0440 protein |
| 1. AA856739 | CUG triplet repeat, RNA |
| 1. T95619 | serine/threonine kinase 17a (apoptosis |
| 1. AA876357 | cofactor required for Sp1 transcriptional activation, subunit 3 (130kD) |
| 1. AI018242 | hypothetical protein FLJ21916 |
| 1. R02575 | hypothetical protein from EUROIMAGE 42353 |
| 1. AA156787 | TEA domain family member 3 |
| 1. AA779888 | TEA domain family member 3 |
| 1. H80012 | hypothetical protein FLJ21865 |
| 1. AA398769 | zinc finger protein 202 |
| 1. AI001010 | DKFZP434H204 protein |
| 1. AA151002 | epithelial protein up |
| 1. W68711 | Homo sapiens clone 23579 mRNA sequence |
| 1. R81201 | KIAA0244 protein |
| 1. AA166695 | tumor necrosis factor (ligand) superfamily, member 13b |
| 1. AA700351 | hypothetical protein |
| 1. N26482 | heparan sulfate 2 |
| 1. AA679939 |  |
| 1. AA910876 |  |
| 1. AA663826 | Homo sapiens cDNA: FLJ21248 fis, clone COL01235 |
| 1. R54846 | fibroblast growth factor receptor 1 (fms |
| 1. AA026624 | hypothetical protein from EUROIMAGE 2107395 |
| 1. AA708940 | leucine zipper |
| 1. T91974 | Homo sapiens cDNA: FLJ22132 fis, clone HEP20459 |
| 1. AA862722 | adaptor |
| 1. AA864791 | hypothetical protein FLJ21313 |
| 1. AA778204 | KIAA0788 protein |
| 1. AA187207 | AD021 protein |
| 1. AA718929 | Homo sapiens mRNA; cDNA DKFZp434L0217 (from clone DKFZp434L0217); partial cds |
| 1. AA775431 | HT014 |
| 1. AA625667 | hypothetical protein DKFZp566D1346 |
| 1. H72093 | hypothetical protein from EUROIMAGE 1034327 |
| 1. AA609959 | Human DNA sequence from clone 1068E13 on chromosome 20p11.21 |
| 1. AA625804 |  |
| 1. AA490846 |  |
| 1. H72756 | pellino (Drosophila) homolog 1 |
| 1. AA883187 | regulator of G |
| 1. N95381 | APG5 (autophagy 5, S. cerevisiae) |
| 1. AA983765 | coronin, actin |
| 1. AA719238 | hypothetical protein DKFZp434H2215 |
| 1. N94746 | hypothetical protein FLJ20758 |
| 1. R26696 | spindlin |
| 1. AA903616 | Human DNA sequence from clone RP5 |
| 1. b |  |
| 1. H13257 | F |
| 1. AA625551 | homeo box A7 |
| 1. AA400198 | KIAA1374 protein |
| 1. H79839 | glycerol |
| 1. AA872309 | Homo sapiens, clone MGC:10702, mRNA, complete cds |
| 1. AA872096 | haemopoietic progenitor homeobox |
| 1. R77783 | protease, serine, 12 (neurotrypsin, motopsin) |
| 1. AA933728 | sec61 homolog |
| 1. AA101971 | Homo sapiens cDNA FLJ10768 fis, clone NT2RP4000150 |
| 1. AA884151 | seven transmembrane domain orphan receptor |
| 1. AA126265 | calnexin |
| 1. AA775952 | KIAA0763 gene product |
| 1. AA431988 | fatty acid amide hydrolase |
| 1. AA488893 | lipase protein |
| 1. R77916 | Homo sapiens mRNA from chromosome 5q21 |
| 1. AA906237 | AA909380 | postmeiotic segregation increased 2 |
| 1. AI014403 | CGI |
| 1. N74650 | formiminotransferase cyclodeaminase |
| 1. AA485137 | hypothetical protein FLJ20071 |
| 1. AA884837 | Homo sapiens cDNA: FLJ22046 fis, clone HEP09276 |
| 1. AA923560 | hypothetical protein FLJ13154 |
| 1. N49922 | proteolipid protein (Pelizaeus |
| 1. AA983362 | Homer, neuronal immediate early gene, 3 |
| 1. AA701081 | KIAA0416 protein |
| 1. N56925 | cyclic AMP |
| 1. N71160 | cytochrome c oxidase subunit VIb |
| 1. R20618 | KIAA1409 protein |
| 1. AI000935 | hypothetical protein FLJ10774; KIAA1709 protein |
| 1. H53025 | calumenin |
| 1. AA626710 | caspase 6, apoptosis |
| 1. AA975680 | Homo sapiens, clone IMAGE:3659680, mRNA, partial cds |
| 1. AI004187 | hypothetical protein |
| 1. AA953644 | vacuolar protein sorting 33B (yeast homolog) |
| 1. T74699 | KIAA0062 protein |
| 1. AA491206 | KIAA0217 protein |
| 1. AA974173 | CGI |
| 1. AA233549 | D component of complement (adipsin) |
| 1. AA773993 | Homo sapiens cDNA FLJ10004 fis, clone HEMBA1000076 |
| 1. N58558 | serine (or cysteine) proteinase inhibitor, clade A (alpha |
| 1. R39697 |  |
| 1. AA936738 | deafness, autosomal dominant 5 |
| 1. AA827468 | Homo sapiens cDNA: FLJ22071 fis, clone HEP11691 |
| 1. AA488185 | L |
| 1. AA772826 | hypothetical protein FLJ13456 |
| 1. AA992906 | KIAA1393 protein |
| 1. AA480859 | pleckstrin homology, Sec7 and coiled/coil domains 1(cytohesin 1) |
| 1. AA476241 | procollagen |
| 1. AA418021 | ICEBERG caspase |
| 1. AA598668 | chimerin (chimaerin) 1 |
| 1. H17022 | DKFZP586F1018 protein |
| 1. AA186873 | Homo sapiens, Similar to RIKEN cDNA 3110001A18 gene, clone MGC:2714, mRNA, complete cds |
| 1. T99639 | KH |
| 1. N30161 | proline |
| 1. AA620859 | sarcospan (Kras oncogene |
| 1. AA873338 | AI733020 | |
| 1. AA886740 | Homo sapiens cDNA FLJ14057 fis, clone HEMBB1000337 |
| 1. AA191479 | SBBI31 protein |
| 1. H91826 | FYN oncogene related to SRC, FGR, YES |
| 1. AA994205 | peptide transporter 3 |
| 1. R59968 | crystallin, mu |
| 1. AA903175 | DNA segment on chromosome 12 (unique) 2489 expressed sequence |
| 1. AA682337 | vav 2 oncogene |
| 1. AA865362 | delta (Drosophila) |
| 1. AA773341 | Dmx |
| 1. AA476277 | hypothetical protein FLJ12750 |
| 1. AA629261 | KIAA1430 protein |
| 1. H51117 | phosphodiesterase IB, calmodulin |
| 1. AA161161 | multiple inositol polyphosphate phosphatase 1 |
| 1. AA520985 | rab3 GTPase |
| 1. AA889785 | nuclear factor I/C (CCAAT |
| 1. AA917854 | KIAA1641 protein |
| 1. AA214392 | SMC2 (structural maintenance of chromosomes 2, yeast) |
| 1. AA992668 | Homo sapiens cDNA: FLJ21771 fis, clone COLF7779 |
| 1. AA679489 | tumor differentially expressed 1 |
| 1. AA702350 | KIAA0442 protein |
| 1. AA278840 | KIAA0210 gene product |
| 1. N71647 | hypothetical protein FLJ10330 |
| 1. H93605 | KIAA0554 protein |
| 1. AI024045 | eukaryotic translation initiation factor 5A2 |
| 1. AI014400 | U6 snRNA |
| 1. AA454947 | A kinase (PRKA) anchor protein 1 |
| 1. AA012939 | Human clone 23801 mRNA sequence |
| 1. N50526 |  |
| 1. AA256532 | insulin |
| 1. AA465214 | DKFZP564N1363 protein |
| 1. H82227 | Homo sapiens, pre |
| 1. AA872505 | Homo sapiens mRNA; cDNA DKFZp434B115 (from clone DKFZp434B115) |
| 1. AA463452 |  |
| 1. AA160695 | KIAA0233 gene product |
| 1. AA490920 | MHC class II transactivator |
| 1. R38031 | peflin |
| 1. AA521482 | hypothetical protein |
| 1. AA455062 | mannosidase, alpha, class 1A, member 2 |
| 1. AA450123 | enolase 2, (gamma, neuronal) |
| 1. AA488996 | spinal cord |
| 1. H72612 | beta tubulin 1, class VI |
| 1. AA705229 | hypothetical protein FLJ12270 |
| 1. AA699331 | hypothetical protein from EUROIMAGE 1967720 |
| 1. AA055114 | phosphoinositide |
| 1. AA600189 | adenosine deaminase, RNA |
| 1. R33030 | glucose regulated protein, 58kD |
| 1. AA451904 | epididymis |
| 1. AA131406 | monokine induced by gamma interferon |
| 1. AA878951 | a disintegrin and metalloproteinase domain 17 (tumor necrosis factor, alpha, converting enzyme) |
| 1. AA679509 | Homo sapiens cDNA: FLJ23067 fis, clone LNG04993 |
| 1. AA454959 | dynein, cytoplasmic, light intermediate polypeptide 2 |
| 1. AA029094 | nuclear receptor subfamily 1, group I, member 3 |
| 1. AA862390 | nudix (nucleoside diphosphate linked moiety X) |
| 1. AA180321 | S164 protein |
| 1. H48096 | TNF receptor |
| 1. AA005382 | granzyme K (serine protease, granzyme 3; tryptase II) |
| 1. H58959 | hypothetical protein FLJ20373 |
| 1. N69204 | chromosome segregation 1 (yeast homolog) |
| 1. R45254 | Homo sapiens mRNA; cDNA DKFZp761M0111 (from clone DKFZp761M0111) |
| 1. AA487265 | KIAA0102 gene product |
| 1. H15567 | KIAA0218 gene product |
| 1. T58146 | MHC class I region ORF |
| 1. AA702802 | azurocidin 1 (cationic antimicrobial protein 37) |
| 1. H71218 | POP7 (processing of precursor, S. cerevisiae) homolog |
| 1. AA994821 | CGI |
| 1. AA143437 | ras homolog gene family, member |
| 1. T96987 | KIAA0929 protein Msx2 interacting nuclear target (MINT) homolog |
| 1. AA858026 | serine (or cysteine) proteinase inhibitor, clade A (alpha |
| 1. AA188233 | DKFZP434K2235 protein |
| 1. R38935 | Homo sapiens clone 25156 mRNA sequence |
| 1. AA460981 | golgi autoantigen, golgin subfamily a, 4 |
| 1. AA879124 | hypothetical protein FLJ00052 |
| 1. AA446565 | S164 protein |
| 1. AA459247 | surfeit 5 |
| 1. R38198 | quinoid dihydropteridine reductase |
| 1. R06106 | transporter protein; system N1 Na+ and H+ |
| 1. AA953036 | hypothetical protein FLJ20154 |
| 1. AA070358 | transketolase (Wernicke |
| 1. H18630 | KIAA0523 protein |
| 1. AA430052 | KIAA1488 protein |
| 1. AI017008 | v |
| 1. H08849 | glutamate receptor, ionotropic, N |
| 1. N51048 | LIS1 |
| 1. H12279 | H12280 | phosphoglucomutase 5 |
| 1. AA668695 | KIAA0716 gene product |
| 1. AA905671 |  |
| 1. AA705308 | fetuin B |
| 1. W72651 | LCAT |
| 1. AA452282 | AD |
| 1. AA074622 | Homo sapiens cDNA: FLJ23567 fis, clone LNG10928 |
| 1. AA703277 | hypothetical protein FLJ11085 |
| 1. N71394 | lymphocyte adaptor protein |
| 1. AA705113 | hydroxysteroid (17 |
| 1. AA961361 | hypothetical protein HRIHFB2072 |
| 1. R34682 | hypothetical protein |
| 1. AA910198 | AI733035 | potassium large conductance calcium |
| 1. AA677535 | ataxin |
| 1. AA676578 | KIAA0692 protein |
| 1. AA889026 |  |
| 1. AA905968 | hypothetical protein PRO2133 |
| 1. H90902 | CDC23 (cell division cycle 23, yeast, homolog) |
| 1. H91146 | integrin |
| 1. AA488899 | KIAA0916 protein |
| 1. AA777229 | retinoid X receptor, alpha |
| 1. W84773 | ATP |
| 1. AA705518 | DKFZP434C245 protein |
| 1. AA488986 | CGI |
| 1. AI023724 | KIAA0160 protein |
| 1. AA400263 | SRY (sex determining region Y) |
| 1. T69304 | TAP binding protein (tapasin) |
| 1. AA235597 | MAD (mothers against decapentaplegic, Drosophila) homolog 6 |
| 1. AA452149 | dual |
| 1. AA188999 |  |

List of 180 Genes which shows up regulation by array I.

| AA454947 | A kinase (PRKA) anchor protein 1 |
| --- | --- |
| AA452282 | AD |
| AA187207 | AD021 protein |
| H21045 | adenosine A1 receptor |
| R89358 | aldo |
| N55484 | aryl hydrocarbon receptor nuclear translocator |
| AA677535 | ataxin |
| W84773 | ATP |
| AA883675 | ATPase, Ca++ transporting, type 2C, member 1 |
| AA504160 | ATPase, H+ transporting, lysosomal (vacuolar proton pump), alpha polypeptide, 70kD, isoform 1 |
| AA702802 | azurocidin 1 (cationic antimicrobial protein 37) |
| AA083385 | BTB (POZ) domain containing 1 |
| AA625850 | butyrate |
| AA858390 | calcyphosine |
| AA704729 | Cas |
| H90902 | CDC23 (cell division cycle 23, yeast, homolog) |
| R53891 | CGI |
| AA994821 | CGI |
| AA488986 | CGI |
| AA598668 | chimerin (chimaerin) 1 |
| H96867 | chromosome 12 open reading frame 4 |
| AA718954 | chromosome 9 open reading frame 11 |
| N69204 | chromosome segregation 1 (yeast homolog) |
| AA876357 | cofactor required for Sp1 transcriptional activation, subunit 3 (130kD) |
| AA705772 | complement component 3 |
| AA856739 | CUG triplet repeat, RNA |
| R22625 | cyclin |
| AA936738 | deafness, autosomal dominant 5 |
| AA705518 | DKFZP434C245 protein |
| AA969787 | DKFZP434F091 protein |
| AI001010 | DKFZP434H204 protein |
| AA188233 | DKFZP434K2235 protein |
| H17022 | DKFZP586F1018 protein |
| AA452149 | dual |
| AA626255 | enoyl Coenzyme A hydratase, short chain, 1, mitochondrial |
| AA151002 | epithelial protein up |
| AI024045 | eukaryotic translation initiation factor 5A2 |
| AA705308 | fetuin B |
| AA884755 | FGFR1 oncogene partner |
| R54846 | fibroblast growth factor receptor 1 (fms |
| AA452840 | fibulin 2 |
| AA192422 | fucosyltransferase 8 (alpha (1,6) fucosyltransferase) |
| T67105 | G protein |
| N34827 | glucuronidase, beta |
| H08849 | glutamate receptor, ionotropic, N |
| AA486570 | glutathione S |
| AA193116 | glycerol |
| R42852 | glycoprotein M6B |
| AA460981 | golgi autoantigen, golgin subfamily a, 4 |
| AA005382 | granzyme K (serine protease, granzyme 3; tryptase II) |
| AA461110 | growth arrest |
| AA919115 | GTPase Rab14 |
| AI017231 | GTT1 protein |
| N26482 | heparan sulfate 2 |
| AA620894 | heterochromatin |
| AA663826 | Homo sapiens cDNA: FLJ21248 fis, clone COL01235 |
| AA701489 | Homo sapiens cDNA: FLJ21429 fis, clone COL04205 |
| AA827468 | Homo sapiens cDNA: FLJ22071 fis, clone HEP11691 |
| N80764 | Homo sapiens cDNA: FLJ23494 fis, clone LNG01885 |
| AA074622 | Homo sapiens cDNA: FLJ23567 fis, clone LNG10928 |
| W68711 | Homo sapiens clone 23579 mRNA sequence |
| AA774824 | Homo sapiens clone 23649 and 23755 unknown mRNA, partial cds |
| AA018114 | Homo sapiens clone 24649 mRNA sequence |
| R38935 | Homo sapiens clone 25156 mRNA sequence |
| AA626281 | Homo sapiens clone CDABP0008 mRNA sequence |
| AI016677 | Homo sapiens clone PP1744 unknown mRNA |
| AA625853 | Homo sapiens MARKL1 mRNA for MAP/microtubule affinity |
| AA707484 | Homo sapiens mRNA; cDNA DKFZp434A1014 (from clone DKFZp434A1014); partial cds |
| AA718929 | Homo sapiens mRNA; cDNA DKFZp434L0217 (from clone DKFZp434L0217); partial cds |
| AA670359 | Homo sapiens mRNA; cDNA DKFZp564B1162 (from clone DKFZp564B1162); complete cds |
| AA045282 | Homo sapiens mRNA; cDNA DKFZp564O0862 (from clone DKFZp564O0862) |
| R45254 | Homo sapiens mRNA; cDNA DKFZp761M0111 (from clone DKFZp761M0111) |
| AA160484 | Homo sapiens PAC clone RP5 |
| H61825 | Homo sapiens spinster |
| AA918089 | Homo sapiens, clone IMAGE:3629896, mRNA, partial cds |
| AA186873 | Homo sapiens, Similar to RIKEN cDNA 3110001A18 gene, clone MGC:2714, mRNA, complete cds |
| AA775431 | HT014 |
| AA012939 | Human clone 23801 mRNA sequence |
| AA609959 | Human DNA sequence from clone 1068E13 on chromosome 20p11.21 |
| AA705113 | hydroxysteroid (17 |
| AA776327 | hypothetical protein |
| AA173423 | hypothetical protein |
| AA700351 | hypothetical protein |
| R34682 | hypothetical protein |
| R33219 | hypothetical protein DKFZp547A023 |
| AA625667 | hypothetical protein DKFZp566D1346 |
| AA776474 | hypothetical protein DKFZp566J091 |
| AA879124 | hypothetical protein FLJ00052 |
| N71647 | hypothetical protein FLJ10330 |
| AA703277 | hypothetical protein FLJ11085 |
| AA778874 | hypothetical protein FLJ11220 |
| AA772826 | hypothetical protein FLJ13456 |
| AA625899 | hypothetical protein FLJ14351 |
| AA953036 | hypothetical protein FLJ20154 |
| AA099360 | hypothetical protein FLJ20285 |
| H58959 | hypothetical protein FLJ20373 |
| H80012 | hypothetical protein FLJ21865 |
| AI018242 | hypothetical protein FLJ21916 |
| AA626878 | hypothetical protein FLJ22477 |
| H72093 | hypothetical protein from EUROIMAGE 1034327 |
| AA026624 | hypothetical protein from EUROIMAGE 2107395 |
| R02575 | hypothetical protein from EUROIMAGE 42353 |
| AA961361 | hypothetical protein HRIHFB2072 |
| AA905968 | hypothetical protein PRO2133 |
| AA418021 | ICEBERG caspase |
| H91146 | integrin |
| T62627 | interferon |
| T99639 | KH |
| AA487265 | KIAA0102 gene product |
| AI023724 | KIAA0160 protein |
| AA278840 | KIAA0210 gene product |
| H15567 | KIAA0218 gene product |
| R81201 | KIAA0244 protein |
| W49494 | KIAA0375 gene product |
| R37938 | KIAA0440 protein |
| H18630 | KIAA0523 protein |
| H93605 | KIAA0554 protein |
| AA862693 | KIAA0804 protein |
| AA488899 | KIAA0916 protein |
| T96987 | KIAA0929 protein Msx2 interacting nuclear target (MINT) homolog |
| AA865147 | KIAA1108 protein |
| AA992906 | KIAA1393 protein |
| AA430052 | KIAA1488 protein |
| AA670270 | KiSS |
| AA488185 | L |
| AA001432 | laminin, alpha 3 (nicein (150kD), kalinin (165kD), BM600 (150kD), epilegrin) |
| W72651 | LCAT |
| N51048 | LIS1 |
| N71394 | lymphocyte adaptor protein |
| AA235597 | MAD (mothers against decapentaplegic, Drosophila) homolog 6 |
| AA151428 | matrix metalloproteinase 23B |
| H28922 | MCF.2 cell line derived transforming sequence |
| AA905896 | melanoma antigen, family A, 2 |
| AA481759 | menage a trois 1 (CAK assembly factor) |
| T58146 | MHC class I region ORF |
| AA464852 | Notch (Drosophila) homolog 4 |
| R22308 | nuclear phosphoprotein similar to S. cerevisiae PWP1 |
| AA488180 | papillary renal cell carcinoma (translocation |
| H12279 | H12280 | phosphoglucomutase 5 |
| AA480859 | pleckstrin homology, Sec7 and coiled/coil domains 1(cytohesin 1) |
| H71218 | POP7 (processing of precursor, S. cerevisiae) homolog |
| AA476241 | procollagen |
| N30161 | proline |
| AA018907 | protein phosphatase 3 (formerly 2B), regulatory subunit B (19kD), alpha isoform (calcineurin B, type I) |
| AA629603 | PTPL1 |
| AA610004 | putative oncogene protein hlc14 |
| R38198 | quinoid dihydropteridine reductase |
| AA143437 | ras homolog gene family, member |
| AA913804 | receptor |
| AA457719 | reticulocalbin 1, EF |
| AA777229 | retinoid X receptor, alpha |
| R63802 | ring finger protein 2 |
| AA446565 | S164 protein |
| AA620859 | sarcospan (Kras oncogene |
| AA858026 | serine (or cysteine) proteinase inhibitor, clade A (alpha |
| T95619 | serine/threonine kinase 17a (apoptosis |
| AA598572 | spleen tyrosine kinase |
| AA400263 | SRY (sex determining region Y) |
| AA459247 | surfeit 5 |
| AA902808 | tankyrase, TRF1 |
| T69304 | TAP binding protein (tapasin) |
| AA156787 | TEA domain family member 3 |
| AA779888 | TEA domain family member 3 |
| AA070358 | transketolase (Wernicke |
| AA999953 | transmembrane protease, serine 4 |
| R06106 | transporter protein; system N1 Na+ and H+ |
| AA166695 | tumor necrosis factor (ligand) superfamily, member 13b |
| AI014400 | U6 snRNA |
| AI017008 | v |
| AA150491 | WAP four |
| AA398769 | zinc finger protein 202 |
| AA629356 |  |
| H73640 |  |
| AA679939 |  |
| AA910876 |  |
| AA873338 | AI733020 |  |
| N50526 |  |
| AA905671 |  |
| AA889026 |  |
| AA188999 |  |

List 134 genes which showed down regulation by array I.

| R72244 | 2' |
| --- | --- |
| AA905362 | ADP |
| AA775447 | alpha |
| T67558 | androgen induced protein |
| N95381 | APG5 (autophagy 5, S. cerevisiae) |
| AA873159 | apolipoprotein C |
| R88535 | ataxin 2 |
| AA481503 | calmodulin 2 (phosphorylase kinase, delta) |
| AA126265 | calnexin |
| H53025 | calumenin |
| AA626710 | caspase 6, apoptosis |
| AA279755 | CD69 antigen (p60, early T |
| AI014403 | CGI |
| N68172 | CGI |
| AA490216 | CK2 interacting protein 1; HQ0024c protein |
| AA865224 | core promoter element binding protein |
| AA983765 | coronin, actin |
| T84491 | CUG triplet repeat, RNA |
| N56925 | cyclic AMP |
| N71160 | cytochrome c oxidase subunit VIb |
| AA918380 | diacylglycerol O |
| AI004779 | DKFZP434A236 protein |
| AA707503 | DKFZP434I092 protein |
| AI024401 | DKFZP434J046 protein |
| AA773341 | Dmx |
| H13257 | F |
| AA431988 | fatty acid amide hydrolase |
| T94279 | fibrinogen, gamma polypeptide |
| N74650 | formiminotransferase cyclodeaminase |
| AA918755 | frequenin (Drosophila) homolog |
| AA256172 | Gardner |
| AI015686 | glioma tumor suppressor candidate region gene 2 |
| T73468 | glutathione S |
| H79839 | glycerol |
| AA872096 | haemopoietic progenitor homeobox |
| AA199881 | heat shock 90kD protein 1, alpha |
| AA999776 | heat shock transcription factor 4 |
| AA625551 | homeo box A7 |
| AA983362 | Homer, neuronal immediate early gene, 3 |
| AA101971 | Homo sapiens cDNA FLJ10768 fis, clone NT2RP4000150 |
| AA884837 | Homo sapiens cDNA: FLJ22046 fis, clone HEP09276 |
| AA459006 | Homo sapiens cDNA: FLJ22063 fis, clone HEP10326 |
| AA907721 | Homo sapiens cDNA: FLJ22097 fis, clone HEP17013 |
| AA863296 | Homo sapiens cDNA: FLJ22448 fis, clone HRC09541 |
| AA663589 | Homo sapiens HSPC302 mRNA, partial cds |
| AA867983 | Homo sapiens mRNA for KIAA1764 protein, partial cds |
| AA708793 | Homo sapiens mRNA for P53TG1 |
| R77916 | Homo sapiens mRNA from chromosome 5q21 |
| AI017640 | Homo sapiens mRNA; cDNA DKFZp434H1235 (from clone DKFZp434H1235); partial cds |
| AA406607 | Homo sapiens mRNA; cDNA DKFZp434I0535 (from clone DKFZp434I0535); partial cds |
| AI004181 | Homo sapiens ubiquitin protein ligase (UBE3B) mRNA, partial cds |
| AA098892 | Homo sapiens, clone IMAGE:2958115, mRNA, partial cds |
| AA975680 | Homo sapiens, clone IMAGE:3659680, mRNA, partial cds |
| AA872309 | Homo sapiens, clone MGC:10702, mRNA, complete cds |
| AA779931 | Homo sapiens, Similar to RIKEN cDNA 1700073K01 gene, clone MGC:12458, mRNA, complete cds |
| AA962376 | HSPC182 protein |
| AA903616 | Human DNA sequence from clone RP5 |
| AI004187 | hypothetical protein |
| R38360 | hypothetical protein |
| AA719238 | hypothetical protein DKFZp434H2215 |
| N53411 | hypothetical protein DKFZp761O0113 |
| AA883397 | hypothetical protein DKFZp762O076 |
| AI000935 | hypothetical protein FLJ10774; KIAA1709 protein |
| AA206614 | hypothetical protein FLJ11175 |
| AA886870 | hypothetical protein FLJ11200 |
| N74593 | hypothetical protein FLJ11848 |
| AA476277 | hypothetical protein FLJ12750 |
| AA923560 | hypothetical protein FLJ13154 |
| AA485137 | hypothetical protein FLJ20071 |
| H66983 | hypothetical protein FLJ20333 |
| AA865924 | hypothetical protein FLJ20464 |
| AA885476 | hypothetical protein FLJ20618 |
| N94746 | hypothetical protein FLJ20758 |
| AA404609 | hypothetical protein FLJ22418 |
| AA682850 | hypothetical protein FLJ22609 |
| AA635186 | IMP (inosine monophosphate) dehydrogenase 2 |
| AA426086 | inactivation escape 2 |
| AA029934 | integrin, alpha V (vitronectin receptor, alpha polypeptide, antigen CD51) |
| AA291389 | interferon |
| AA701081 | KIAA0416 protein |
| AA670383 | KIAA0551 protein |
| N71435 | KIAA0701 protein |
| AA775952 | KIAA0763 gene product |
| H41237 | KIAA0921 protein |
| AA676756 | KIAA1031 protein |
| AA919136 | KIAA1271 protein |
| AA400198 | KIAA1374 protein |
| R20618 | KIAA1409 protein |
| AA488893 | lipase protein |
| W72329 | lymphotoxin alpha (TNF superfamily, member 1) |
| AA045458 | M |
| AA504113 | M |
| AA872274 | mannosyl (alpha |
| AA278296 | mitogen |
| AI017072 | myoneurin |
| AA428321 | N |
| H85094 | nucleosome assembly protein 1 |
| N73548 | olfactory receptor, family 2, subfamily I, member 6 |
| AA704985 | phosphorylase kinase, alpha 2 (liver) |
| AA430032 | pituitary tumor |
| AA906237 | AA909380 | postmeiotic segregation increased 2 |
| R77783 | protease, serine, 12 (neurotrypsin, motopsin) |
| N49922 | proteolipid protein (Pelizaeus |
| T95805 | putative mitochondrial outer membrane protein import receptor |
| AA683102 | RAD21 (S. pombe) homolog |
| AA933728 | sec61 homolog |
| N71049 | serine (or cysteine) proteinase inhibitor, clade A (alpha |
| R10382 | serine (or cysteine) proteinase inhibitor, clade A (alpha |
| AA845156 | serine protease inhibitor, Kazal type 1 |
| AA884151 | seven transmembrane domain orphan receptor |
| AA453813 | sialyltransferase 4C (beta |
| T97303 | signal sequence receptor, alpha (translocon |
| AA894634 | slit (Drosophila) homolog 2 |
| AA775980 | solute carrier family 1 (glial high affinity glutamate transporter), member 2 |
| AA772989 | Sp4 transcription factor |
| R26696 | spindlin |
| AA679000 | Splicing factor, arginine/serine |
| AA683351 | suppressor of white apricot homolog 2 |
| AA719156 | testes |
| H59861 | thrombomodulin |
| AA955007 | transaldolase 1 |
| AA088701 | transmembrane, prostate androgen induced RNA |
| AA953644 | vacuolar protein sorting 33B (yeast homolog) |
| AA856703 | vacuolar protein sorting 35 (yeast homolog) |
| AA864204 | x 001 protein |
| AA134771 | zinc finger protein 24 (KOX 17) |
| AA629022 |  |
| AA777043 |  |
| AA780740 |  |
| AA918907 |  |
| AI004349 |  |
| b |  |
| N/A |  |
| N48620 |  |
